# Profiling cancer-related gene mutations in oral squamous cell carcinoma from Japanese patients by targeted amplicon sequencing

**DOI:** 10.18632/oncotarget.19262

**Published:** 2017-07-15

**Authors:** Takafumi Nakagaki, Miyuki Tamura, Kenta Kobashi, Ryota Koyama, Hisayo Fukushima, Tomoko Ohashi, Masashi Idogawa, Kazuhiro Ogi, Hiroyoshi Hiratsuka, Takashi Tokino, Yasushi Sasaki

**Affiliations:** ^1^ Department of Medical Genome Sciences, Research Institute for Frontier Medicine, Sapporo Medical University, Sapporo, Japan; ^2^ Department of Oral Surgery, Sapporo Medical University School of Medicine, Sapporo, Japan

**Keywords:** OSCC, next-generation sequencing, Ion Torrent, somatic mutations, copy number variations

## Abstract

Somatic mutation analysis is a standard practice in the study of human cancers to identify mutations that cause therapeutic sensitization and resistance. We performed comprehensive genomic analyses that used PCR target enrichment and next-generation sequencing on Ion Proton semiconductor sequencers. Forty-seven oral squamous cell carcinoma (OSCC) samples and their corresponding noncancerous tissues were used for multiplex PCR amplification to obtain targeted coverage of the entire coding regions of 409 cancer-related genes (covered regions: 95.4% of total, 1.69 megabases of target sequence). The number of somatic mutations in 47 patients with OSCC ranged from 1 to 20 with a mean of 7.60. The most frequent mutations were in *TP53* (61.7%), *NOTCH1* (25.5%), *CDKN2A* (19.1%), *SYNE1* (14.9%), *PIK3CA* (10.6%), *ROS1* (10.6%), and *TAF1L* (10.6%). We also detected copy number variations (CNVs) in the segments of the genome that could be duplicated or deleted from deep sequencing data. Pathway assessment showed that the somatic aberrations within OSCC genomes are mainly involved in several important pathways, including cell cycle regulation and RTK–MAPK-PI3K. This study may enable better selection of therapies and deliver improved outcomes for OSCC patients when combined with clinical diagnostics.

## INTRODUCTION

Head and neck squamous cell carcinoma (HNSCC) is the sixth most common malignancy in developed countries, representing nearly 2.4% of all malignancies [1]. Oral squamous cell carcinoma (OSCC), a subset of HNSCC, accounts for > 90% of malignancies that affect the oral cavity. The etiology of OSCC is well established and mainly involves the use of tobacco and alcohol. However, there are no sensitive biomarkers to improve the early detection of oral cancers. Many published studies have evaluated markers of the cell cycle, the immune response, apoptosis, and angiogenesis as well as adhesion- and matrix degradation-related molecules. The poor prognosis of OSCC patients is associated with the overexpression of anti-apoptotic genes and the deregulation of p53 function [2–4], both of which may contribute to chemotherapy resistance. However, the precise molecular mechanism underlying the resistance to chemotherapy displayed by recurrent OSCC remains largely unknown.

Since unique mutations have been observed in individual human cancer samples, the identification and characterization of the molecular alterations underlying individual cancer patients are critical to developing more effective, personalized therapies. For example, next-generation sequencing (NGS) technologies have revolutionized cancer genomics research by providing a comprehensive method of detecting somatic cancer genome alterations, including point mutations, insertions, deletions, and copy number variations (CNVs). Investigators have also uncovered several critical genes and pathways important in the tumorigenesis of HNSCC, including *TP53*, *CDKN2A*, *PIK3CA*, and *CCND1* [5]. To date, three studies have been published in which whole exome or whole genome sequencing was performed using high-throughput NGS in HNSCC tumors [6–8]. Additional NGS-mediated discoveries included mutations in cell differentiation pathways, particularly in *NOTCH* and *FBXW7* [6]. The India Project Team of the International Cancer Genome Consortium published an exome sequencing study of 50 Indian patients with OSCC [9]. Recent analysis of smokeless tobacco-associated OSCC in Arabian patients revealed altered pathways not previously implicated in OSCC, such as Oncostatin-M signaling and AP-1 and C-MYB transcription networks [10]. However, these molecular alterations do not fully recapitulate the pathogenesis of OSCC. In addition, no study to date has focused on the genetic characterization of Japanese OSCC patients.

NGS technologies have several advantages over classical Sanger sequencing, including the ability to generate large quantities of DNA sequence information in a single run to detect genetic mosaicism in depth [11]. However, the routine usage of these technologies leaves us with several limitations, such as the cost of entry and long processing times. Recently, the Ion Torrent sequencing technology based on semiconductor sequencing [12] has substantially circumvented many of these issues. This platform, which has a very low input DNA requirement and is compatible with FFPE samples, makes DNA sequencing cheaper, faster, and more reliable. In the present study, 9 OSCC cell lines and surgically resected OSCC tissues from 47 Japanese patients were sequenced for mutations in the coding regions of 409 cancer-related genes using a semiconductor-based sequencing platform. This targeted next-generation sequencing had significant advantages over the classical molecular methods used to perform high-throughput sequencing in clinical laboratories.

## RESULTS

### Targeted amplicon sequencing of human OSCC

We performed semi-conductor sequencing of all exons of 409 cancer-related genes in 9 OSCC cell lines as well as 47 OSCC tumors (14 FFPE and 33 frozen samples) and 38 matched normal controls (26 peripheral white blood cells and 12 normal adjacent tissues) using the Ion Ampliseq Comprehensive Cancer Panel (Supplementary Figure 1, Thermo Fisher Scientific, Waltham, MA). The sequencing overview, including reads, coverage, and uniformity of the read coverage distribution, is shown in Supplementary Tables 1 and 2. Each sample underwent an average 6.2 million sequencing reads after quality filtering. A mean coverage depth of 426.1, 306.2, 423.2, and 406.3 reads per base were observed for cell lines, FFPE samples, frozen samples, and peripheral white blood cells, respectively. The most common mutations in OSCCs were C>T transitions (50.2%), which is a process attributed to the normal cellular event of the deamination of 5-methylcytosine. C>T transitions were also the most common mutations found in HNSCC and esophageal squamous cell carcinoma (ESCC) in previous whole exome sequencing studies (Supplementary Figure 2). Importantly, the second most frequent mutations in OSCC were C>A transversions. It is well known that the carcinogen benzo[*a*]pyrene in tobacco predominantly induces C>A transversions [13], which Song et al. reported to be the most common mutation in lung squamous cell carcinoma (LSCC) [14].

**Table 1:** Characteristics of OSCC patients

Parameters		No. (n = 47)
Gender	Female	21
	Male	26
Age (y)	≤ 49	4
	50~59	9
	60~69	16
	70~79	14
	80~89	4
Site of primary tumor	Tongue	24
	Gingiva	13
	Mouth floor	8
	Other	2
Lymph node metastasis	Negative	26
	Positive	21
Tumor size (cm)	< 4	35
	≥ 4	12
TNM stage	I	11
	II	13
	III	3
	IV	20
Smoking	Yes	24
	No	23
Drinking	Yes	28
	No	19

Validations by Sanger sequencing were performed randomly after NGS analysis. In total, 19 out of 19 single-nucleotide variations (SNVs) and 11 out of 11 insertions and deletions (InDels) (100%) could be confirmed (Supplementary Table 3 and Supplementary Figure 3), indicating very low technical bias.

### Identification of somatic mutations in 9 OSCC cell lines

**Figure 1:**
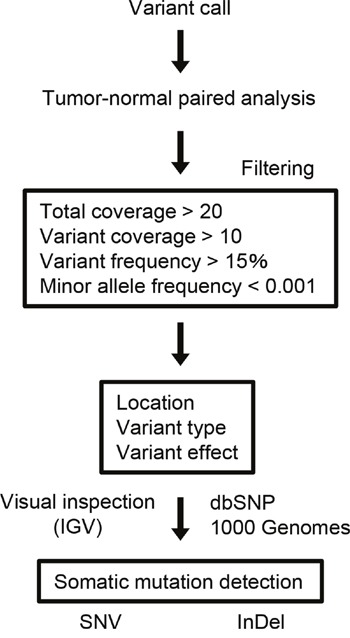
Data analysis pipeline Workflow to identify somatic SNVs and InDels from the sequencing data.

The procedure of somatic mutation detection is shown in Figure 1. For cell lines, known germline variants were filtered out using the dbSNP database (dbSNP version 132) and 6,515 previously published normal exomes [15]. *TP53* mutations, which were predicted to confer loss of function, were noted in all cell lines (10 mutations), including 2 frameshifts and 6 missense, 1 nonsense, and 1 splice site mutations. We also observed 3 nonsynonymous and 1 splice site mutations in *CDKN2A*, 3 nonsynonymous mutations in *SMAD4*, 2 in *NOTCH1*, and 2 in *PIK3CA* (Supplementary Table 4). The Cancer Cell Line Encyclopedia (CCLE, http://www.broadinstitute.org/ccle) includes over 1,000 cancer cell lines and has the mutational statuses of cancer-related genes. The mutations in *TP53, CDKN2A, PIK3CA*, and *SMAD4* detected in HSC2, HSC3, HSC4, and SCC-25 cells were consistent with the CCLE mutation data.

### Identification of frequent somatic mutations in 409 cancer-related genes in OSCC

Somatic mutations were identified using a tumor-normal analysis in which the germline variants were subtracted from the tumor variants. The sequencing results for the tumor DNA were compared with those obtained from either matching peripheral white blood cells (n = 26), normal adjacent tissues (n = 12), or control sequence data within the Ion Reporter software (n = 9, for the patients who did not have DNA from matched normal controls).

**Figure 2:**
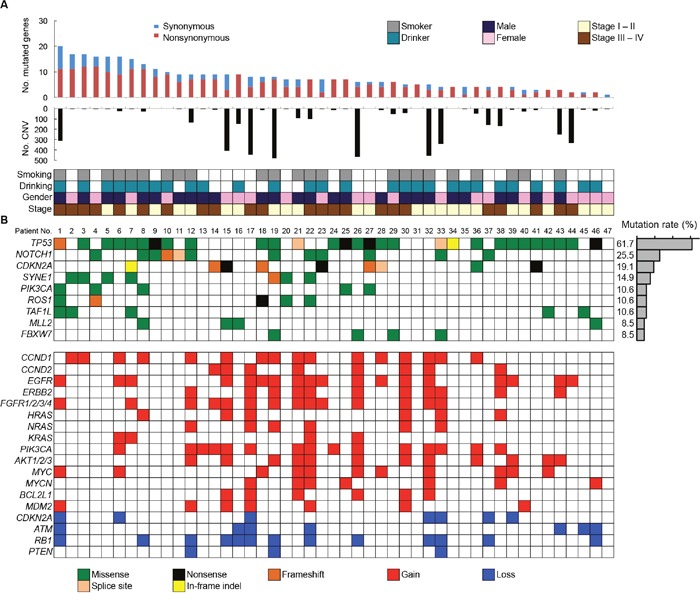
Summary of somatic mutations and CNVs across 47 OSCC samples **(A)** The numbers of synonymous and nonsynonymous mutations (1st panel) and CNV regions (2nd panel) of each examined case are shown. In the bottom panel, the first row indicates smoking status, the second row indicates drinking status, the third row indicates gender, and the fourth row indicates cancer stage. Columns correspond to the examined cases. **(B)** Upper panels: Significantly mutated genes colored by the type of mutations and their mutational frequency. Lower panels: Significant CNVs detected in OSCC samples.

All SNVs and InDels detected by bioinformatics analysis underwent visual inspection using the Integrative Genomics Viewer (IGV) for confirmation (examples in Supplementary Figure 4). In total, we identified 357 variants (127 genes) in 47 patients with OSCC (Supplementary Table 5). The mean somatic mutation frequency was 7.60 mutations per OSCC patient (range 1-20, Figure 2A), resulting in a mean mutation rate of 4.50 mutations/Mb. We also identified a mean of 5.49 nonsynonymous mutations (range 0-12) per sample (FFPE, 4.50; frozen, 5.91). This mutation frequency was more than twice as high as the rate seen in HNSCC [7, 9, 16], suggesting that SNVs in OSCC may be concentrated on cancer-related genes. The result also suggested that this panel could effectively detect somatic mutations in cancer tissues. There is an association between smoking behavior and an increased number of nonsynonymous mutations (smokers, 6.3 vs. nonsmokers, 4.6; P < 0.05). The 9 most commonly mutated genes in OSCC tumors are depicted in Figure 2B. *TP53* is the most frequently mutated gene in OSCC tumors [61.7% (29 of 47 cases), 31 mutations]. Of the *TP53* mutations, 22 (71.0%) were predicted to be missense mutations, 5 (16.1%) were predicted to be nonsense mutations, 2 (6.9%) were predicted to be splice site mutations, 1 (3.5%) was predicted to be a frameshift deletion, and 1 (3.5%) was predicted to be an in-frame deletion. In addition, the majority of *TP53* mutations (83.9%, 26/31) are localized in the DNA binding domain of the protein (residues 100-300, Supplementary Figure 5). *TP53* mutations were significantly correlated with a smoking habit in OSCC patients (P < 0.05).

**Figure 3:**
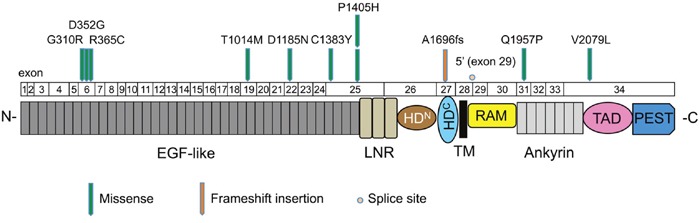
Mutation distribution in the exons and functional domains of NOTCH1 Each arrowhead represents a nonsynonymous mutation in an individual tumor. Individual exons are represented as numbered boxes. Conserved domains were mapped from UniProt. EGF-like, epidermal growth factor-like; LNR, Lin12/Notch repeats; HD^N^, heterodimerization–N terminal; HD^C^, heterodimerization–C terminal; TM, transmembrane; RAM, Rbp-associated molecule; Ankyrin, CDC10/ankyrin domain; TAD, transactivation domain; and PEST, a region rich in proline (P), glutamate (E), serine (S), and threonine (T).

In total, 25.5% of the cases examined showed mutations in *NOTCH1*, which was the second most commonly mutated gene in our study, and included 10 missense mutations (9 sites), 1 frameshift insertion, and 1 splice site mutation (Supplementary Table 6). The mutation distribution across the NOTCH1 functional domains is shown in Figure 3. Interestingly, eight mutations (66.7%) were located in the EGF-like domains of the NOTCH1 extracellular region. An amino acid sequence comparison of mutation sites among species revealed that six of the nine *NOTCH1* missense mutations (G310R, D352G, R365C, T1014M, C1383Y, and Q1957P) were on highly conserved residues among vertebrate *Notch*1 orthologs, suggesting significant functional effects of *NOTCH1* mutations in the pathogenesis of OSCC (Supplementary Figure 6). Furthermore, four of nine *NOTCH1* missense mutations (G310R, D352G, D1185N, and Q1957P) were on conserved residues among all four human *Notch* paralogs (Supplementary Figure 7). The significance of *NOTCH1* mutations has also been confirmed by the observation that the frequency of mutations in *NOTCH1* is much higher than the frequency of mutations in its paralogs *NOTCH2* and *NOTCH4* (Supplementary Table 5).

Other frequently mutated genes in OSCC include *CDKN2A* (19.1%), *SYNE1* (14.9%), *PIK3CA* (10.6%), *ROS1* (10.6%), *TAF1L* (10.6%), *MLL2* (8.5%), and *FBXW7* (8.5%). All the nonsense and missense mutations in *CDKN2A* and missense mutations in *PIK3CA* are listed in the COSMIC database, suggesting the functional effects of the mutations (Supplementary Table 7). *CDKN2A* genes exhibited nonsense and frameshift mutations, indicating its tumor suppressor roles in OSCC. Four out of five tumors with mutated *PIK3CA* involved the cervical lymph nodes (Supplementary Table 7), suggesting that *PIK3CA* mutations may be a late event in the progression of OSCC.

### Target amplicon sequencing detects CNVs

We also detected CNVs in the segments of the genome that could be duplicated or deleted from sequencing data (example are shown in Supplementary Figures 8 and 9). A number of candidate driver genes were identified from the CNVs (Figure 2B, Lower panel). The genes most frequently affected were *EGFR* (gain in 38.3%) and *CCND1* (gain in 34.0%), followed by *PIK3CA* (gain in 31.9%), *RB1* (loss in 27.7%), *FGFR1/2/3/4* (gain in 30.0%), *AKT1/2/3* (gain in 25.5%), *ERBB2* (gain in 23.4%), *MYC* (gain in 23.4%), *CCND2* (gain in 17.0%), *MYCN* (gain in 14.9%), *ATM* (loss in 14.9%), *MDM2* (gain in 14.9%), *CDKN2A* (loss in 14.9%), *NRAS* (gain in 12.8%), *HRAS* (gain in 12.8%), *BCL2L1* (gain in 12.8%), and *KRAS* (gain in 8.5%). We validated 100% (16/16), 100% (8/8), 83.3% (5/6), 83.3% (5/6) and 100% (5/5) of the OSCC tissues that showed CNV gain for *CCND1, CCND2, HRAS, NRAS* and *KRAS* respectively in quantitative PCR copy number analysis (Supplementary Figure 10). In addition, CNV status of *CCND1* and *CCND2* was associated with mRNA expression levels in OSCC cell lines (Supplementary Figure 11).

### Signaling pathways altered in patients with OSCC

**Figure 4:**
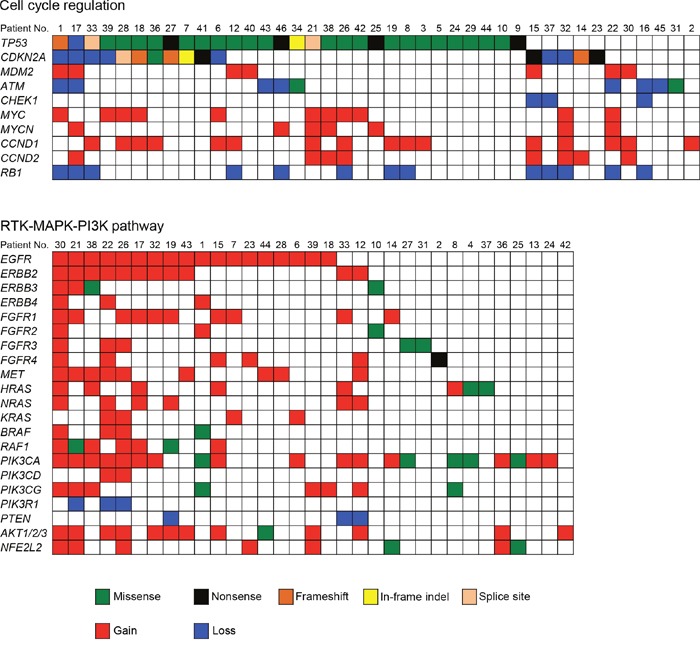
Genetic alterations identified by the sequencing of 409 cancer-related genes across 47 OSCCs impact cell cycle regulation and the RTK-MAPK-PI3K pathways Each column denotes an individual tumor, and each row represents a gene. Somatic alterations are colored by the type of the event. A major proportion of patients with OSCC harbored alterations in one or more components in the pathways of cell cycle regulation (87.2%) and RTK-MAPK-PI3K signaling (70.2%).

To obtain a more comprehensive understanding of the molecular lesions in Japanese OSCC, we conducted a literature review to identify the pathways that were genetically deregulated (Figure 4). Cell cycle regulatory components constituted the most frequently disrupted category (87.2%, 41 of 47 cases), including mutations and CNV loss in *TP53* (63.8%), *CDKN2A* (34.0%), and *ATM* (19.1%); there were also CNV gains in *CCND1* (34.0%), *MYC* (23.4%), *CCND2* (17.0%), *MDM2* (14.9%), and *MYCN* (14.9%) and CNV losses in *RB1* (27.7%) and *CHEK1* (6.4%). The other frequently altered pathways included receptor tyrosine kinase, mitogen-activated protein kinase, and the phosphoinositide 3-kinase (RTK/MAPK/PI3K) pathway (disrupted in 70.2%, 33 of 47 cases). Several receptor tyrosine kinases (*EGFR*, *ERBB2-4, FGFR1-4*, and *MET*) and their downstream signal transducers (*PIK3CA, NRAS, HRAS, KRAS*, and *AKT1*) are targets in this pathway (Figure 4).

### Association of genetic alterations with clinical outcomes

We then analyzed potential association between actionable driver mutations (*TP53, NOTCH1*, *CDKN2A*, and *PIK3CA*) and clinical outcomes (Supplementary Figure 12A-12D). Univariate survival analysis revealed that mutations in *NOTCH1* and *PIK3CA* were found to be associated with worse overall survival in OSCC patients (*P* = 0.0054 and *P* = 0.0162, respectively). In contrast, none of these associations were found to be statistically significant on *TP53 and CDKN2A* mutations. Interestingly, RTK/MAPK/PI3K pathway alteration correlated to poor survival (Supplementary Figure 12E), suggesting that RTK/MAPK/PI3K pathway is potentially a therapeutic target in this disease. As expected, the clinical outcome of OSCC patients is strongly influenced by the stage of disease (Supplementary Figure 12F).

## DISCUSSION

The advent of NGS has resulted in the identification of actionable genomic alterations, which are capable of providing more accurate information for the treatment of cancer patients [16, 17]. Whole genome and whole exome sequencings are still too expensive for routine use and involve difficult data analysis. Therefore, cost-effective alternatives are important. In our cohort, all tumors were shown by the panel-based approach to carry at least one somatic mutation, suggesting that this panel has the potential to obtain robust data and detect genetic events in tumors. A total of 357 somatic mutations were identified in 47 OSCC patients. The frequency of *TP53* mutations is fairly consistent with the frequency found in previous studies of OSCC (62%-73%) [8, 9]. Importantly, the *TP53* gene was frequently mutated in early stage OSCC (stages I and II, 62.5%, 15/24 in Figure 2). In line with our observations, previous studies have also shown that *TP53* mutations occur in early stage HNSCC as well as in oral premalignant lesions, which are often identified as leukoplakia [18–20]. These observations indicate that TP53 is a key molecule for the progression of oral tumorigenesis. The *TP53* gene is the most commonly mutated gene in human malignancies and has many important biological functions, including the control of the cell cycle checkpoint [21]. Moreover, we identified the cell cycle regulation pathway as the most significantly altered pathway (Figure 4).

Pathway analysis of the genomics data also indicated that 70.2% of samples had genes altered in the RTK/MAPK/PI3K pathway (Figure 4). RTKs of the ERBB family (EGFR and ERBB2-4) are involved in the development and progression of epithelial tumors. Induction of RTK phosphorylation by ligand binding activates downstream pathways such as RAS-RAF-MEK–ERK–MAPK [22], which function in modulating normal cell growth and survival. Thus, these signaling pathways have been proposed as attractive targets for the development of molecular therapeutics for treating human cancer [23, 24]. Tyrosine kinase inhibitors, such as erlotinib and gefitinib, are currently frontline therapeutics in *EGFR* mutant non-small cell lung cancer patients. By contrast, cetuximab, a monoclonal antibody directed against EGFR, shows a clinical benefit in cancer patients who overexpress EGFR in a manner independent of *EGFR* mutations. We found that *RTKs* and *MET* were found to be amplified or to have somatic mutations in OSCC. The introduction of cetuximab resulted in a modest improvement in the survival of HNSCC patients [25, 26]; therefore, the determination of how to effectively treat tumors with therapeutic targets in this pathway should be considered a priority. We also observed a co-occurrence of alterations involving the RTK/MAPK/PI3K pathway in almost half of the tumor samples (Figure 4). Importantly, Kaplan–Meier analysis showed that RTK/MAPK/PI3K pathway alteration was associated with a poorer prognosis in OSCC patients. These results indicate that simultaneous inhibition of this pathway may be required to achieve therapeutic benefit.

The Notch signaling pathway is thought to play important roles in regulating normal cell differentiation and survival, especially in multiple stages of metazoan development [27]. Notch signaling dysregulation is implicated in a number of human diseases, including cancers and developmental disorders [28]. Interestingly, Notch signaling has both oncogenic and tumor-suppressive roles depending on the cellular context [29]. The oncogenic activity of this pathway has been observed in a number of hematopoietic cancers [30]. By contrast, loss-of-function *NOTCH1* mutations are relatively common in HNSCC, lung SCC, and breast cancer [6, 31, 32]. We found that *NOTCH1* was the second most commonly mutated gene in Japanese patients with OSCC. The frequency of *NOTCH1* mutations in this study (25.5%, Figure 2B) was higher than the frequency observed in the 530 HNSCC tumors of the TCGA cohort (17.8%) (http://www.cbioportal.org/study?id=hnsc_tcga#summary). In addition, *NOTCH1* had a significantly higher rate of mutation in Chinese OSCC patients (43.1%-48.5%) [33, 34], suggesting that *NOTCH1* mutations may be a characteristic feature of OSCC patients of Asian descent. Of the 12 nonsynonymous mutations, one was a frameshift, 10 were missense mutations, and one was a splice site mutation, which are all generally predicted to be loss-of-function mutations. Many of these missense mutations occurred at or near important domains such as ligand-binding domains (EGF-like domain) (Figure 3). Moreover, we found that most missense mutations observed in *NOTCH1* could affect highly conserved amino acids among species (Supplementary Figure 6) and were predicted to be deleterious according to the PolyPhen-2 tool, which predicts the possible impact of an amino acid substitution (Supplementary Table 6). We also found mutations in three other genes related to the Notch pathway, including *FBXW7* (4 cases), *NFE2L2* (2 cases), and *KEAP1* (1 case) (Supplementary Table 5), implicating Notch signaling pathways in the pathogenesis of OSCC.

The other recurrently mutated candidate genes included the synaptic nuclear envelope protein 1 (*SYNE1*) (14.9%), TATA-box binding protein associated factor 1 like (*TAF1L*) (10.6%), receptor tyrosine kinase (ROS1) (10.6%), and histone methyltransferase *MLL2/KMT2D* (8.5%) (Figure 2). Among them, *MLL2* is frequently mutated in HNSCC, with a mutation rate of 11%-18% [7, 8]. In addition to *MLL2*, we identified *MLL* mutations in two cases and *MLL3* mutations in three cases (Supplementary Table 5). These frequent mutations in *MLL* family members indicate the critical roles of chromatin/histone modifiers in OSCC. We also found 108 genes that were mutated in one or two cases, which followed a classic long tail distribution of less-frequently mutated genes and highlighted the heterogeneous and complex nature of the disease.

Recent advances in genomic sequencing technologies have caused a paradigm shift in the ways cancer is treated; precision medicine is becoming a reality. Increasing numbers of molecular targeting drugs are under development or have entered into clinical trials [35]. The broad application of NGS for actionable therapeutic target detection relies on several aspects, including the ability to use FFPE materials. In the present study, however, we found that the number of mutations in FFPE was lower than that in frozen samples (4.50 vs. 5.91). Sequence data statistics showed a decreased percentage on-target reads and uniformity for FFPE compared to frozen samples (Supplementary Tables 1 and 2), suggesting that sample preservation could play an important role in the use of tumor tissue for cancer sequencing. The limit of this study is the absence of any relevant targetable mutation among the analyzed genes. We recently performed whole exome sequencing of DNA from 14 OSCC samples and matched normal DNA. As the results, we detected recurrent mutations in the genes not covered in this study, such as *CASP8, EPHA2* and *FAT1* (data not shown). We believe that faster and more cost-effective genomic profiling that can be achieved via the use of an organ-specific gene panel will facilitate the implementation of tailored and personalized therapies in the near future.

## MATERIALS AND METHODS

### Ethics

This study was approved by the Institutional Review Boards of Sapporo Medical University (reference number 26-25 by Sapporo Medical University's Ethics Committee). All patients provided written informed consent. The study was conducted in accordance with the ethical principles of the Declaration of Helsinki.

### Cell lines and tumor specimens

The 9 OSCC cell lines (HSC2, HSC3, HSC4, SKN3, SCC25, HOC119, MON2, SAS, and Ca9-22) used in this study were obtained from the Japanese Collection of Research Bioresources (Tokyo, Japan). Forty-seven patients with OSCC were diagnosed and treated at the Oral Surgery Department of Sapporo Medical University from 2008 to 2015. Hematoxylin and eosin-stained slides were reexamined to confirm the original diagnosis. A list of the tumor samples and their clinical characteristics are shown in Table 1.

### DNA preparation

DNA was extracted from the cell lines, fresh frozen tissue, and peripheral blood samples using the QIAamp DNA Mini kit (Qiagen GmbH, Hilden, Germany) following the manufacturer's instructions. DNA was also isolated from FFPE sections using the QIAamp DNA FFPE Tissue kit (Qiagen). The TaqMan RNase P Detection Reagents kit (Thermo Fisher Scientific) was used to quantify purified DNA.

### Semiconductor-based next-generation sequencing

DNA (40 ng) was used for multiplex PCR amplification with an Ion Ampliseq Comprehensive Cancer Panel, enabling the targeted coverage of all exons of 409 cancer-related genes (covered regions = 95.4% of total). The 15,992 amplicons obtained represented more than 1.69 megabases of target sequence. Library preparation and sequencing with the Ion Torrent sequencer were performed as previously described [36–38]. The templates were sequenced after emulsion PCR with 6-8 samples per Ion PI chip using the Ion PI HI-Q Chef kit (Thermo Fisher Scientific).

### Identification of somatic mutations and CNVs

Human genome build 19 (hg19) was used as a reference. Alignment to the hg19 genome and sequencing read count were performed in Torrent Suite version 5.0 (Thermo Fisher Scientific). Somatic mutations (point mutations, insertions, and deletions) were detected using statistical approaches in tumor and normal samples from the Ion Reporter software 5.0 tumor-normal workflow (Thermo Fisher Scientific). When matched normal controls were not available, the control sequence data provided by Thermo Fisher Scientific was used as a control. A sequencing coverage of 20 x and a minimum variant frequency of 15% of the total number of distinct tags were used as cutoffs. Mutations were called if they occurred in < 0.1% of reads in the normal control and were absent from dbSNP as well as the 1000 Genomes Project database. The IGV software (http://www.broadinstitute.org/igv) was used to filter out possible strand-specific errors, such as a mutation that was only detected in the forward or reverse DNA strand but not in both strands. The SIFT, Polyphen-2, and Grantham scores were used to estimate evolutionary conservation and the effects of an amino acid substitution on the structure and function of the protein. CNV detection was also performed by the Ion Reporter software using an algorithm based on a Hidden Markov Model. Recurrent genomic regions with CNVs were identified using copy numbers greater than 3 and less than 1 for gains and losses, respectively.

## SUPPLEMENTARY MATERIALS FIGURES AND TABLES
















