# Supplementary material for: Profiling cancer-related gene mutations in oral squamous cell carcinoma from Japanese patients by targeted amplicon sequencing
Source: Oncotarget. 2017 Jul 15;8(35):59113–22. doi: 10.18632/oncotarget.19262 (PMC5601718; doi:10.18632/oncotarget.19262)
Supplement: Supplementary file 1 [file oncotarget-08-59113-s001.pdf]

Profiling cancer-related gene mutations in oral squamous cell carcinoma from Japanese patients by targeted amplicon sequencing

SUPPLEMENTARY MATERIALS

ABL1	BTK	DDR2	FLT1	ITGA9	MLH1	PAK3	RALGDS	TCF12
ABL2	BUB1B	DEK	FLT3	ITGB2	MLL	PAX5	RARA	TCF3
ACVR2A	CARD11	DICER1	FLT4	ITGB3	MLL2	PAX7	RB1	TCF7L1
ADAMTS20	CASC5	DNMT3A	FN1	JAK1	MLL3	PALB2	RECQL4	TCF7L2
AFF1	CBL	DPYD	FOXL2	JAK2	MLLT10	PARP1	REL	TCL1A
AFF3	CCND1	DST	FOXO1	JAK3	MMP2	PAX3	RET	TET1
AKAP9	CCND2	EGFR	FOXO3	JUN	MN1	PAX8	RHOH	TET2
AKT1	CCNE1	EML4	FOXP1	KAT6A	MPL	PBRM1	RNASEL	TFE3
AKT2	CD79A	EP300	FOXP4	KAT6B	MRE11A	PBX1	RNF2	TGFBR2
AKT3	CD79B	EP400	FZR1	KDM5C	MSH2	PDE4DIP	RNF213	TGM7
ALK	CDC73	EPHA3	G6PD	KDM6A	MSH6	PDGFB	ROS1	THBS1
APC	CDH1	EPHA7	GATA1	KDR	MTOR	PDGFRA	RPS6KA2	TIMP3
AR	CDH11	EPHB1	GATA2	KEAP1	MTR	PDGFRB	RRM1	TLR4
ARID1A	CDH2	EPHB4	GATA3	KIT	MTRR	PER1	RUNX1	TLX1
ARID2	CDH20	EPHB6	GDNF	KLF6	MUC1	PGAP3	RUNX1T1	TNFAIP3
ARNT	CDH5	ERBB2	GNA11	KRAS	MUTYH	PHOX2B	SAMD9	TNFRSF14
ASXL1	CDK12	ERBB3	GNAQ	LAMP1	MYB	PIK3C2B	SBDS	TNK2
ATF1	CDK4	ERBB4	GNAS	LCK	MYC	PIK3CA	SDHA	TOP1
ATM	CDK6	ERCC1	GPR124	LIFR	MYCL1	PIK3CB	SDHB	TP53
ATR	CDK8	ERCC2	GRM8	LPHN3	MYCN	PIK3CD	SDHC	TPR
ATRX	CDKN2A	ERCC3	GUCY1A2	POT1	MYD88	PIK3CG	SDHD	TRIM24
AURKA	CDKN2B	ERCC4	HCAR1	LPP	MYH11	PIK3R1	SEPT9	TRIM33
AURKB	CDKN2C	ERCC5	HIF1A	LRP1B	MYH9	PIK3R2	SETD2	TRIP11
AURKC	CEBPA	ERG	HLF	LTF	NBN	PIM1	SF3B1	TRRAP
AXL	CHEK1	ESR1	HNF1A	LTK	NCOA1	PKHD1	SGK1	TSC1
BAI3	CHEK2	ETS1	HOOK3	MAF	NCOA2	PLAG1	SH2D1A	TSC2
BAP1	CIC	ETV1	HRAS	MAFB	NCOA4	PLCG1	SMAD2	TSHR
BCL10	CKS1B	ETV4	HSP90AA1	MAGEA1	NF1	PLEKHG5	SMAD4	UBR5
BCL11A	CMPK1	EXT1	HSP90AB1	MAGI1	NF2	PML	SMARCA4	UGT1A1
BCL11B	COL1A1	EXT2	ICK	MALT1	NFE2L2	PMS1	SMARCB1	USP9X
BCL2	CRBN	EZH2	IDH1	MAML2	NFKB1	PMS2	SMO	VHL
BCL2L1	CREB1	FAM123B	IDH2	MAP2K1	NFKB2	POU5F1	SMUG1	WAS
BCL2L2	CREBBP	FANCA	IGF1R	MAP2K2	NIN	PPARG	SOCS1	WHSC1
BCL3	CRKL	FANCC	IGF2	MAP2K4	NKX2-1	PPP2R1A	SOX11	WRN
BCL6	CRTC1	FANCD2	IGF2R	MAP3K7	NLRP1	PRDM1	SOX2	WT1
BCL9	CSF1R	FANCF	IKBKB	MAPK1	NOTCH1	PRKAR1A	SRC	XPA
BCR	CSMD3	FANCG	IKBKE	MAPK8	NOTCH2	PRKDC	SSX1	XPC
BIRC2	CTNNA1	FAS	IKZF1	MARK1	NOTCH4	PSIP1	STK11	XPO1
BIRC3	CTNNB1	FBXW7	IL2	MARK4	NPM1	PTCH1	STK36	XRCC2
BIRC5	CYLD	FGFR1	IL21R	MBD1	NRAS	PTEN	SUFU	ZNF384
BLM	CYP2C19	FGFR2	IL6ST	MCL1	NSD1	PTGS2	SYK	ZNF521
BLNK	CYP2D6	FGFR3	IL7R	MDM2	NTRK1	PTPN11	SYNE1	
BMPR1A	DAXX	FGFR4	ING4	MDM4	NTRK3	PTPRD	TAF1	
BRAF	DCC	FH	IRF4	MEN1	NUMA1	PTPRT	TAF1L	
BRD3	DDB2	FLCN	IRS2	MET	NUP214	RAD50	TAL1	
BRIP1	DDIT3	FLI1	ITGA10	MITF	NUP98	RAF1	TBX22	

Supplementary Figure 1: Ion Ampliseq Comprehensive Cancer Panel gene list.

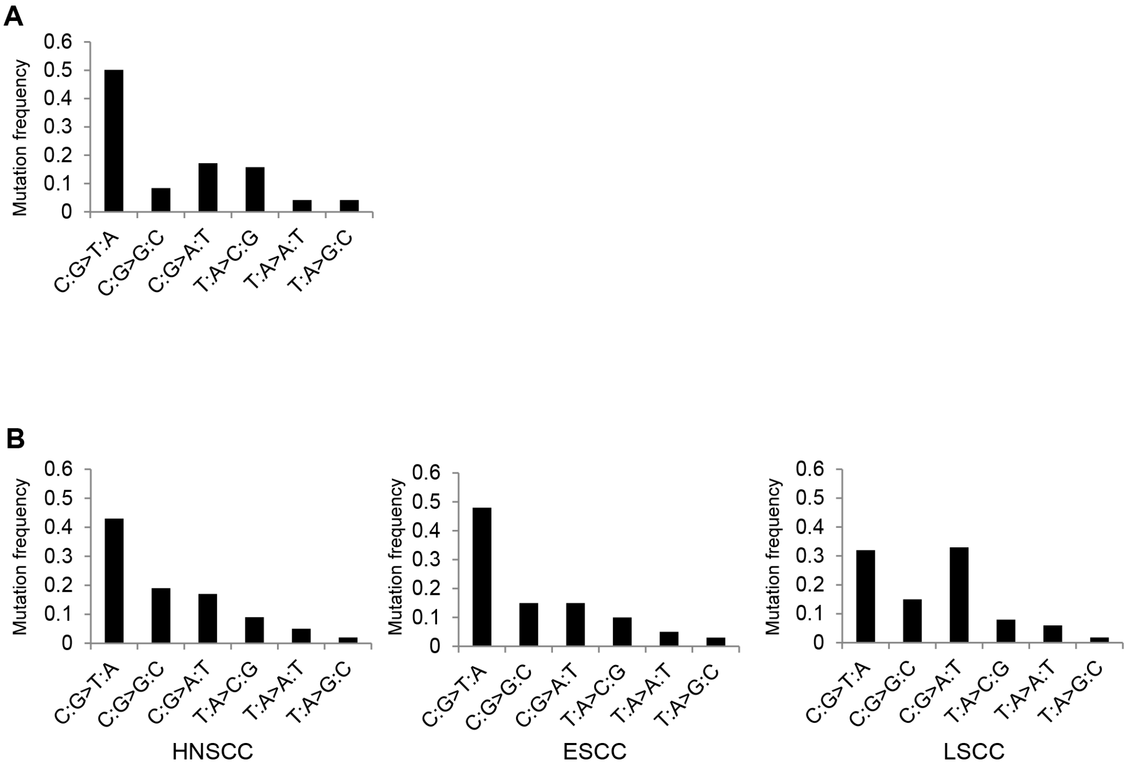

Supplementary Figure 2: Mutation spectrum of single nucleotide substitutions. **(A)** The major nucleotide change was a C/G>T/A transition in this study. The second most frequent mutation was a C/G>A/T transversion. **(B)** Spectrum of single nucleotide substitutions in the exome-sequencing studies of head and neck (HNSCC), esophageal (ESCC), and lung squamous cell carcinoma (LSCC) [1] (Song Y et al. Nature 2014; 509:91-95).

SUPPLEMENTARY REFERENCE

1. Song Y, Li L, Ou Y, Gao Z, Li E, Li X, Zhang W, Wang J, Xu L, Zhou Y, Ma X, Liu L, Zhao Z, et al. Identification of genomic alterations in oesophageal squamous cell cancer. Nature. 2014; 509:91-5.

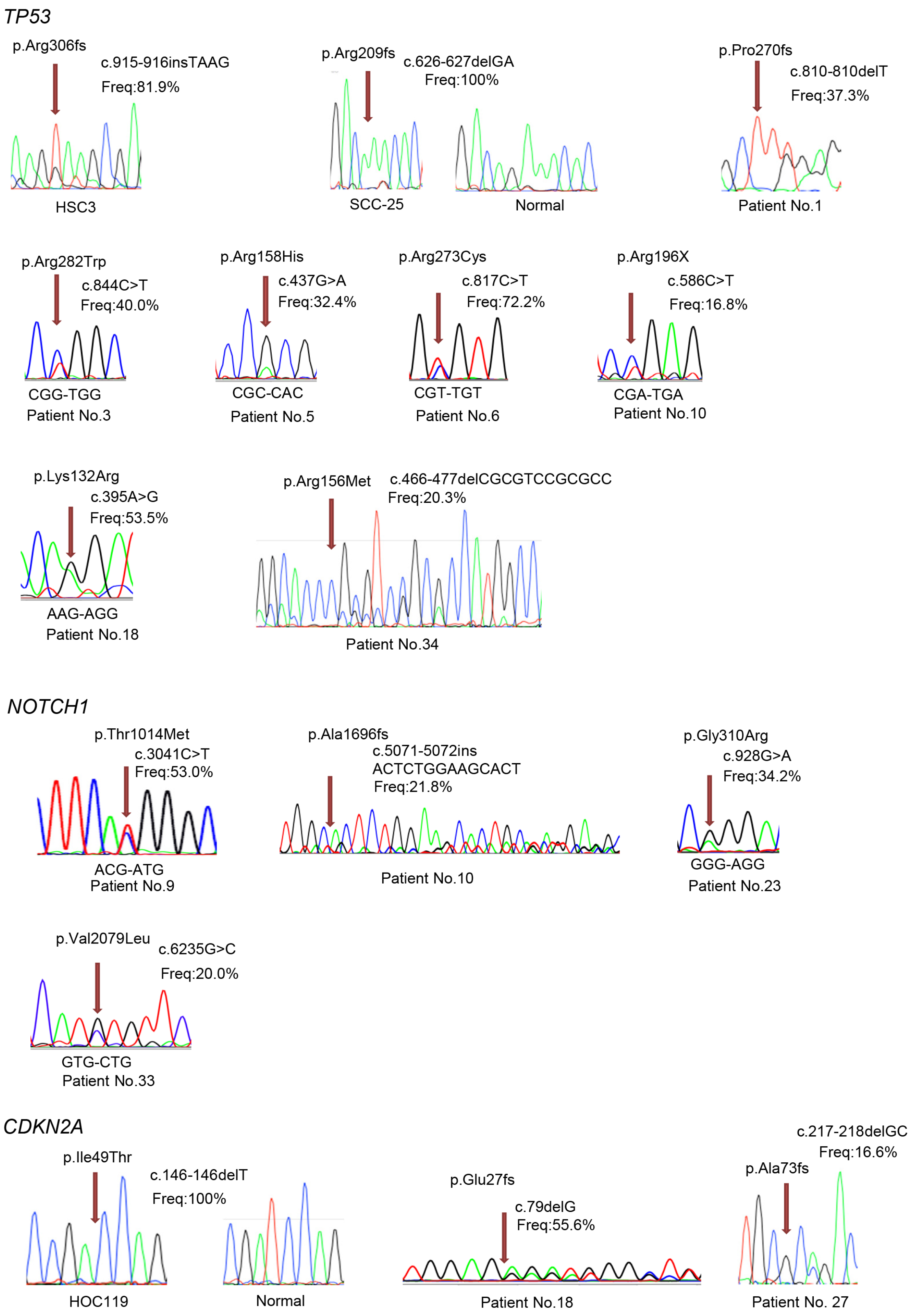
TP53
p.Arg306fs
c.915-916insTAAG
Freq:81.9%
HSC3
p.Arg209fs
c.626-627delGA
Freq:100%
SCC-25
Normal
p.Pro270fs
c.810-810delT
Freq:37.3%
Patient No.1
p.Arg282Trp
c.844C>T
Freq:40.0%
CGG-TGG
Patient No.3
p.Arg158His
c.437G>A
Freq:32.4%
CGC-CAC
Patient No.5
p.Arg273Cys
c.817C>T
Freq:72.2%
CGT-TGT
Patient No.6
p.Arg196X
c.586C>T
Freq:16.8%
CGA-TGA
Patient No.10
p.Lys132Arg
c.395A>G
Freq:53.5%
AAG-AGG
Patient No.18
p.Arg156Met
c.466-477delCGCGTCCGCGCC
Freq:20.3%
Patient No.34
NOTCH1
p.Thr1014Met
c.3041C>T
Freq:53.0%
ACG-ATG
Patient No.9
p.Ala1696fs
c.5071-5072ins
ACTCTGGAAGCACT
Freq:21.8%
Patient No.10
p.Gly310Arg
c.928G>A
Freq:34.2%
GGG-AGG
Patient No.23
p.Val2079Leu
c.6235G>C
Freq:20.0%
GTG-CTG
Patient No.33
CDKN2A
p.Ile49Thr
c.146-146delT
Freq:100%
HOC119
Normal
p.Glu27fs
c.79delG
Freq:55.6%
Patient No.18
c.217-218delGC
Freq:16.6%
p.Ala73fs
Patient No. 27

(*Continued*)

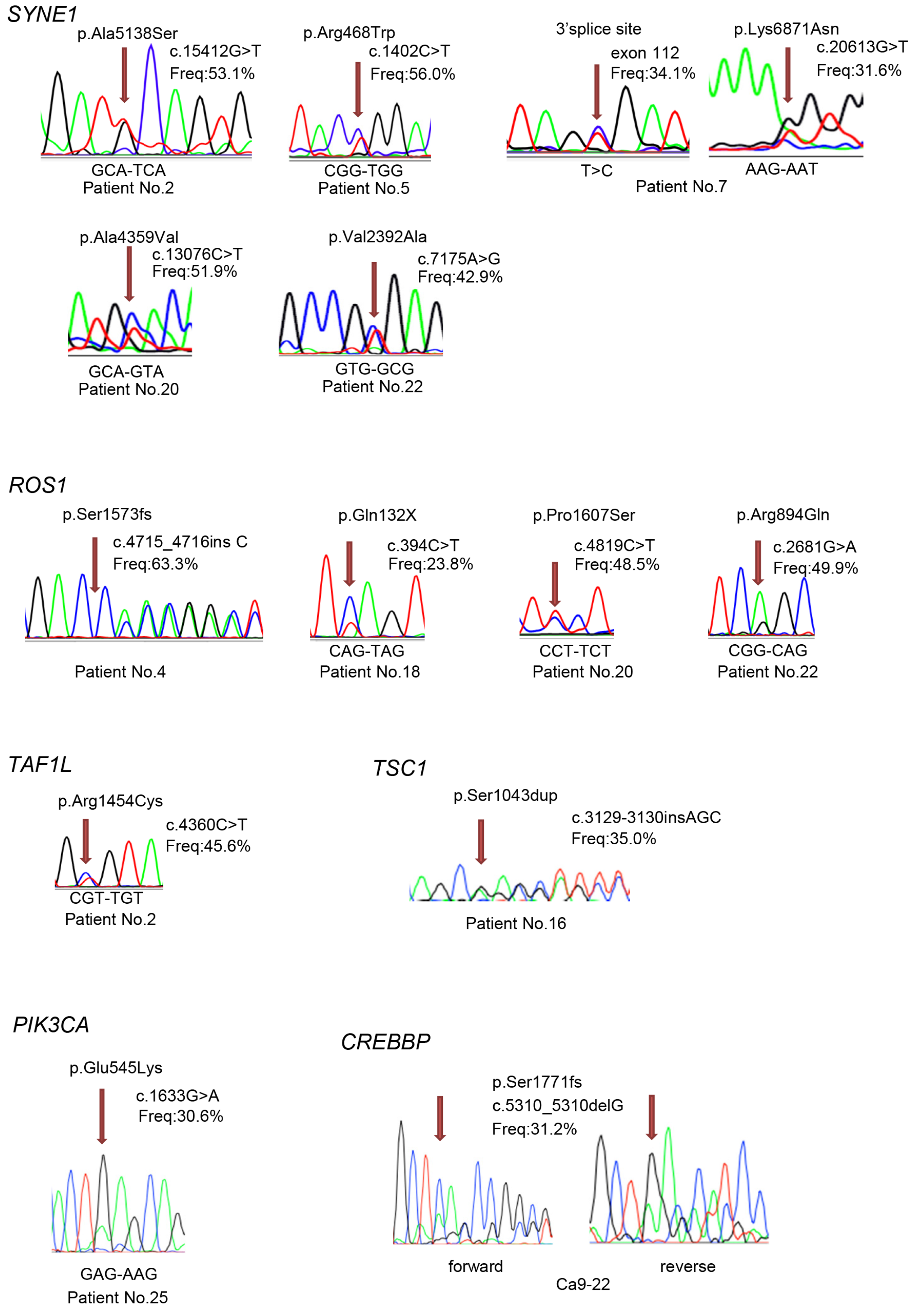

Supplementary Figure 3: Verification of identified mutations by Sanger sequencing. Sanger sequencing traces for 30 variants (19 SNVs and 11 InDels) are shown. The position of the called variant is indicated by an arrow. The variant effect, cDNA position, and variant frequency are also given.

A

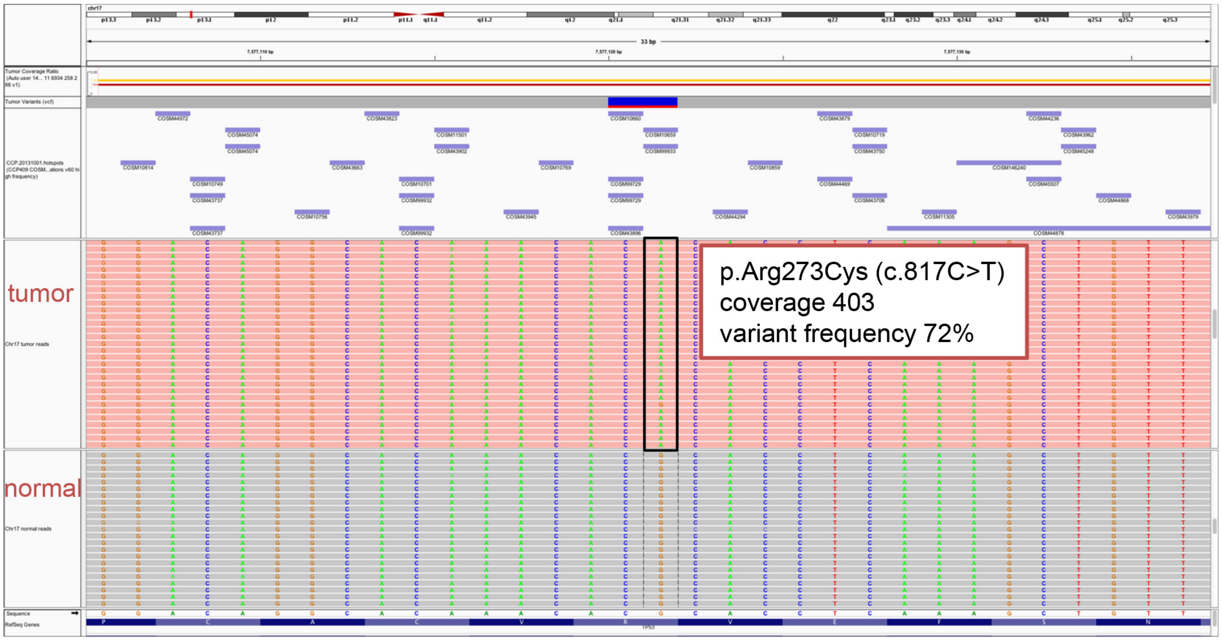

B

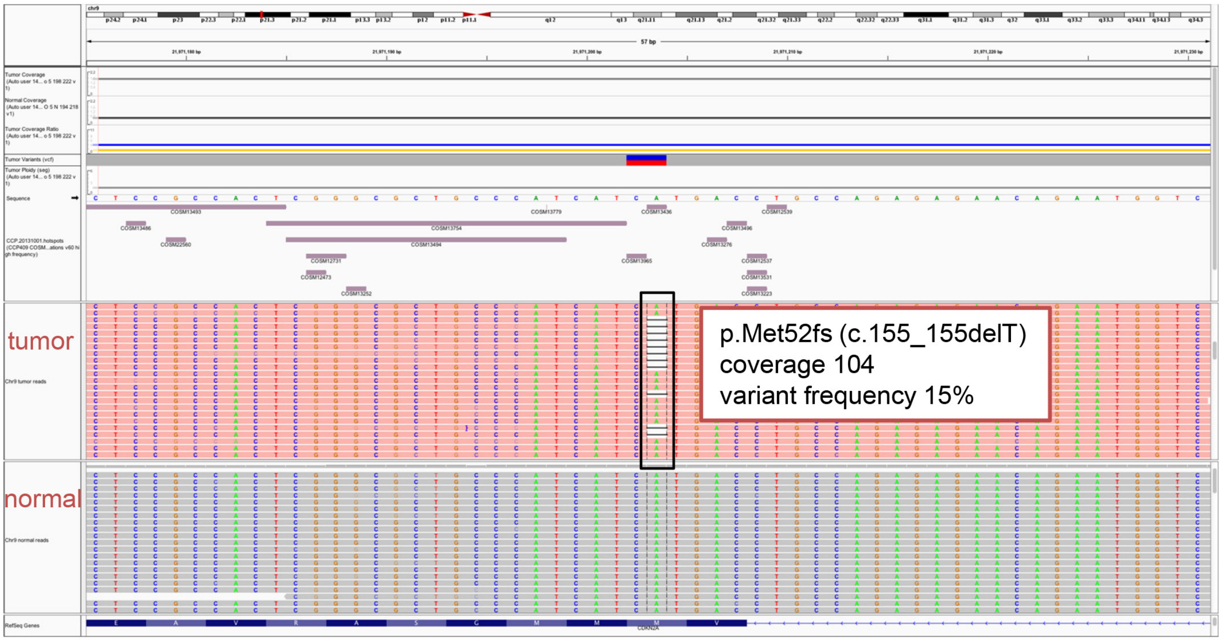

Supplementary Figure 4: Examples of SNVs and InDels detected by NGS. Two types of nonsynonymous mutations from OSCC patients as shown by the IGV. **(A)** A missense mutation detected in exon 8 of the *TP53*gene (p.Arg273Cys, c.817C>T) in patient No. 6. **(B)** A 1-bp deletion detected in exon 2 of the *CDKN2A* gene (c.155_155delT) in patient No. 14.

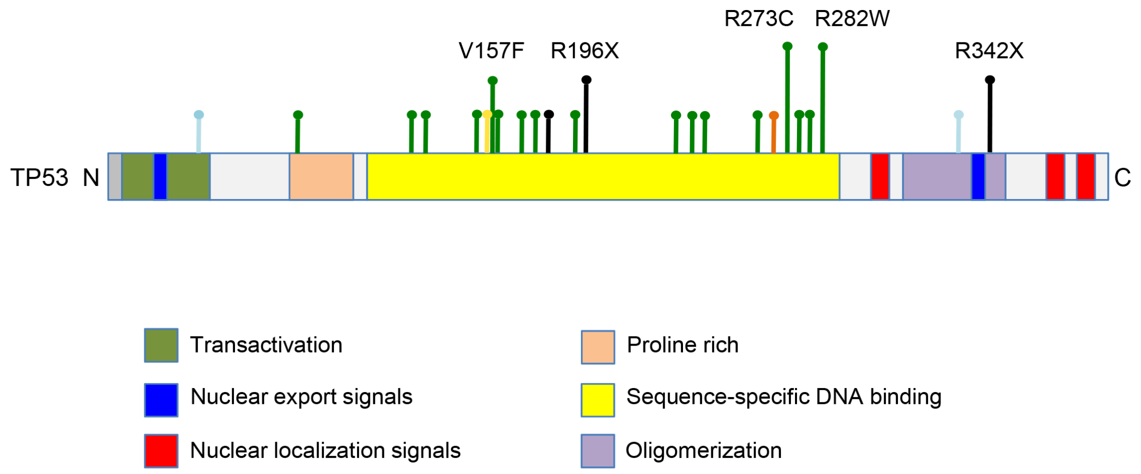

Supplementary Figure 5: Mutation distribution in the function domains of *TP53* in OSCC tumors. Any position with a mutation contains a circle (green, missense mutation; black, nonsense mutation; yellow, in-frame deletion; orange, frameshift deletion; and blue, splice site mutation), and the length of the line depends on the number of mutations detected at that codon. The colored boxes are specific functional domains. Above the lollipops, the frequent variants are annotated as the amino-acid change at that specific site.

Patient No.23

```
HUMAN   305 A C Q N G G T C H N T 315
MACMU   305 A C Q N G G T C H N T 315
MOUSE   305 A C Q N G G T C H N T 315
CHICK   308 A C Q N G G T C H N N 318
ANOCA   310 A C Q N G G T C H N T 319
XENLA   304 A C Q N G G T C H N T 314
DANRE   304 A C Q N G G T C H N T 314
 BILA   945 N Y P D N S E C S W K 955
```

Gly310Arg

Patient No.4

```
 347 G A T C H D R V A S F 357
 347 G A T C H D R V A - - 355
 347 G A T C H D R V A - - 355
 350 G A T C H D R V A - - 358
 351 G A T C H D R V A S F 361
 346 G A T C H D R V A - - 354
 346 G A T C H D R V A - - 354
1010 G A T C H D R V K N I 1020
```

Asp352Gly

Patient No.37

```
HUMAN   360 E C P H G R T G L L C 370
MACMU   361 - C P H G R T G L L C 370
MOUSE   361 - C P H G R T G L L C 370
CHICK   364 - C P H G R T G L L C 373
ANOCA   364 E C P H G R T G L L C 374
XENLA   360 - C P H G R T G L L C 369
DANRE   360 - C P H G R T G - - - 266
 BILA  1029 S S V N S R A S G - - 1037
```

Arg365Cys

Patient No.9

```
1009 C P P G F T G S Y C Q 1019
1009 C P P G F T G S Y C Q 1019
1009 C P P G F T G S Y C Q 1019
1012 C P S G F T G S Y C E 1022
1013 C P P G F T G I Y C E 1023
1008 C P P G F T G S Y C Q 1018
1007 C L P G F T G I Y C Q 1017
1906 - - - - - - - - D A G 1909
```

Thr1014Met

Patient No.12

```
HUMAN  1180 C S E E I D E C L S H 1190
MACMU  1180 C S E E I D E C L S H 1190
MOUSE  1180 C S E E I N E C L S Q 1190
CHICK  1183 C S E E I N E C L S H 1193
ANOCA  1184 C S E E I N E C L S H 1194
XENLA  1179 C S E E I N E C L S H 1189
DANRE  1178 C S K E I N E C L S Q 1188
 BILA  2152 C E I - D D W C N E F 2161
```

Asp1185Asn

Patient No.27

```
1378 F T G P E C Q F P A S 1388
1378 F T G P E C Q F P A S 1388
1378 F T G P E C Q F P A S 1388
1381 F T G P E C Q Y P A S 1391
1382 F T G P E C E F P S S 1392
1377 Y T G A T C Q Y P V I 1387
1376 F S G H E C Q T R M D 1386
2513 F Y G R Y - M T V D P 2522
```

Cys1383Tyr

Patient No.8

Patient No.19

```
HUMAN  1400 Q G T C E P T S - - E 1408
MACMU  1400 Q G T C E P T S - - E 1408
MOUSE  1400 Q G T C E P T S - - E 1408
CHICK  1402 G G T C E F L S D - A 1411
ANOCA  1403 Q G T C Q F A K E P P 1413
XENLA  1398 G G T C Q F F A - - E 1406
DANRE  1397 G G T C Q P I S - - D 1405
 BILA  2533 N G N C S I D D D D D 2543
```

Pro1405His

Patient No.22

```
1952 A D A N I Q D N M G R 1962
1952 A D A N I Q D N M G R 1962
1942 A D A N I Q D N M G R 1952
1955 A D A N I Q D N M G R 1965
1958 A D A N I Q D H M G K 1968
1949 A D A N I Q D N M G R 1959
1939 A D A N I Q D N M G R 1949
3154 T Q T Y H F E Q F G E 3164
```

Gln1957Pro

Patient No.33

```
HUMAN  2074 Y E T A K V L L D H F 2084
MACMU  2074 Y E T A K V L L D H F 2084
MOUSE  2064 Y E T A K V L L D H F 2074
CHICK  2077 Y E T A K V L L D H F 2087
ANOCA  1987 - - - - - - - - - - - 1987
XENLA  2071 Y E T A K V L L D H F 2081
DANRE  2062 Y E T A K V L L D H F 2072
 BILA  3373 T E I - - - - - G K Y 3378
```

Val2079Leu

Supplementary Figure 6: Detected *NOTCH1* mutation sites are well conserved between species. Sequence homology of Notch1 mutation sites from different species is shown. HUMAN, *Homo sapiens*; MACMU, monkey; MOUSE, mouse; CHICK, chicken; ANOCA, lizard; XENLA, frog; DANRE, zebrafish; and BILA, *Trichinella*.

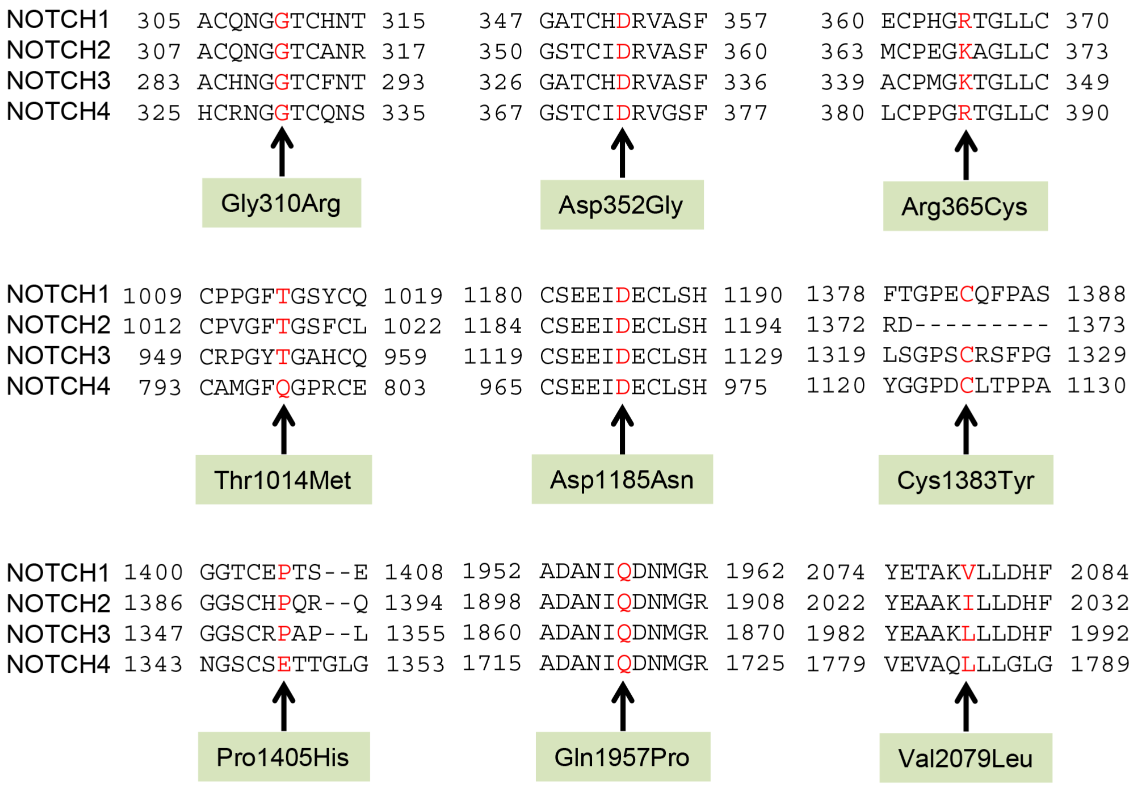

Supplementary Figure 7: Detected *NOTCH1* mutation sites are well conserved throughout the Notch family. Sequence homology of *NOTCH1* mutation sites with three human paralogs (*NOTCH2*, *NOTCH3*, and *NOTCH4*). The alignment was generated using the UniProt workflow.

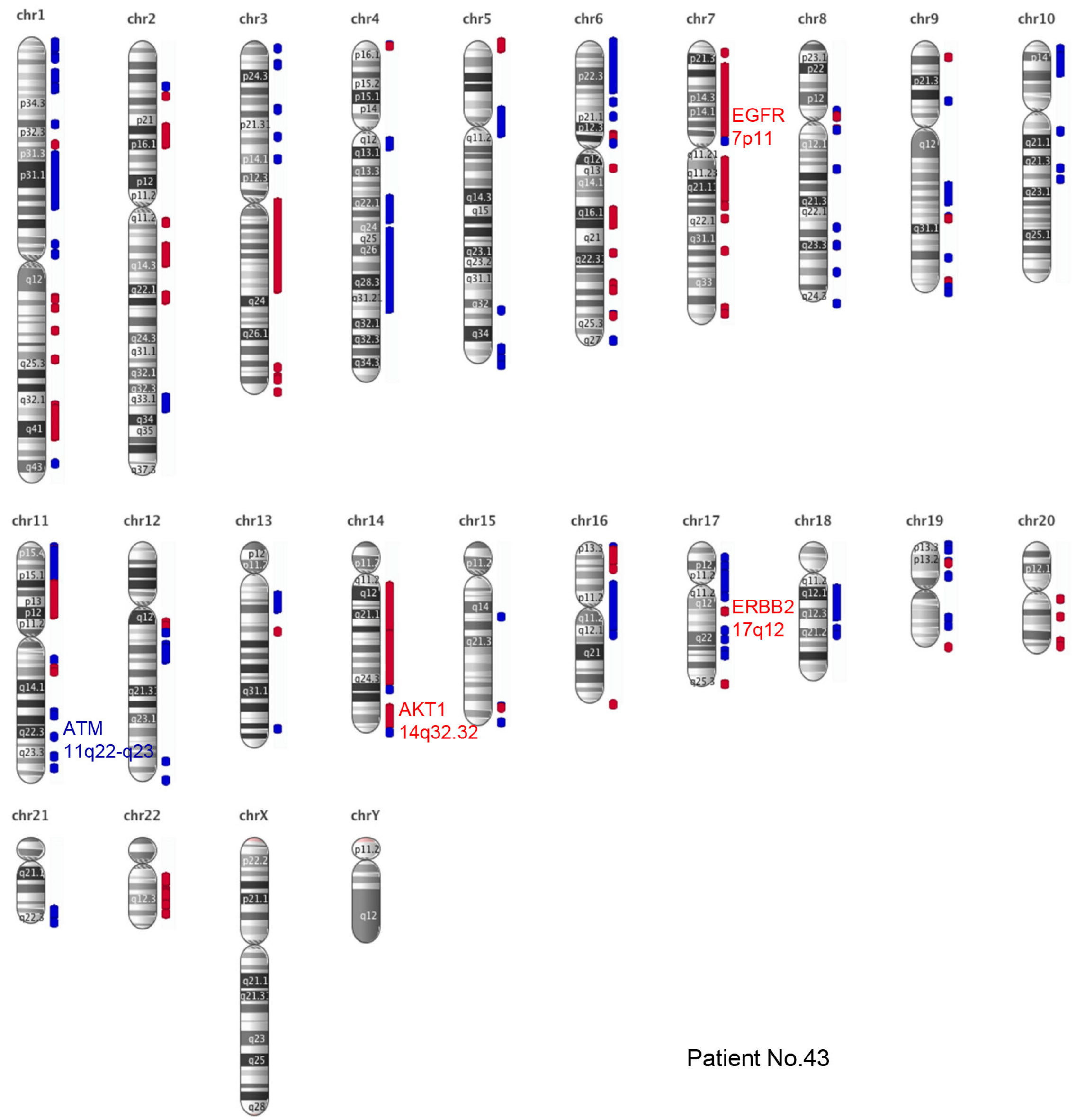

Supplementary Figure 8: Example of an OSCC case with CNVs. Visualization of CNVs over the entire genome in the karyotype view. The decreased and increased copy numbers are indicated in blue and red, respectively.

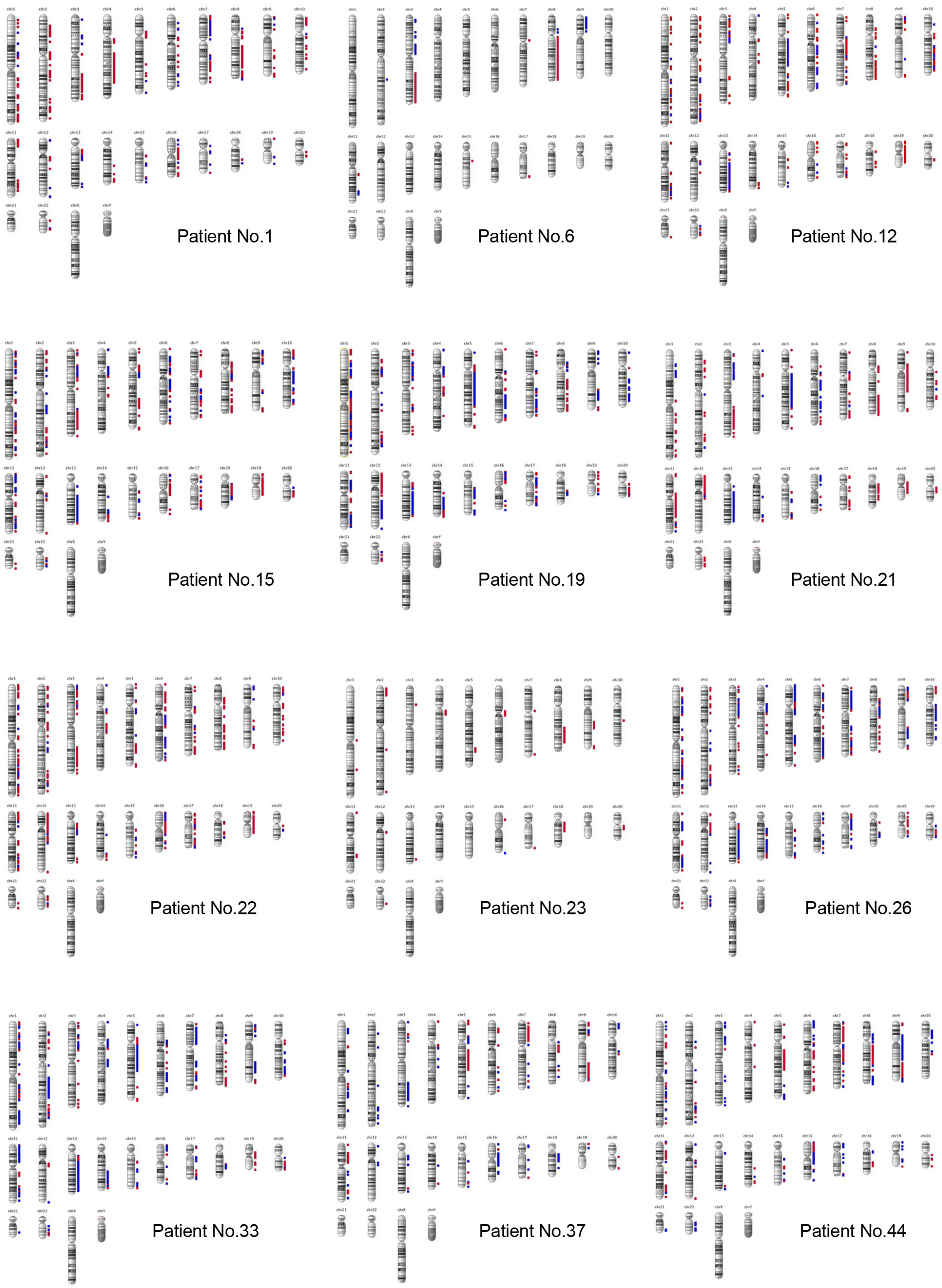

Supplementary Figure 9: Examples of OSCC cases with CNVs. Visualization of CNVs over the entire genome in the karyotype view. The decreased and increased copy numbers are indicated in blue and red, respectively.

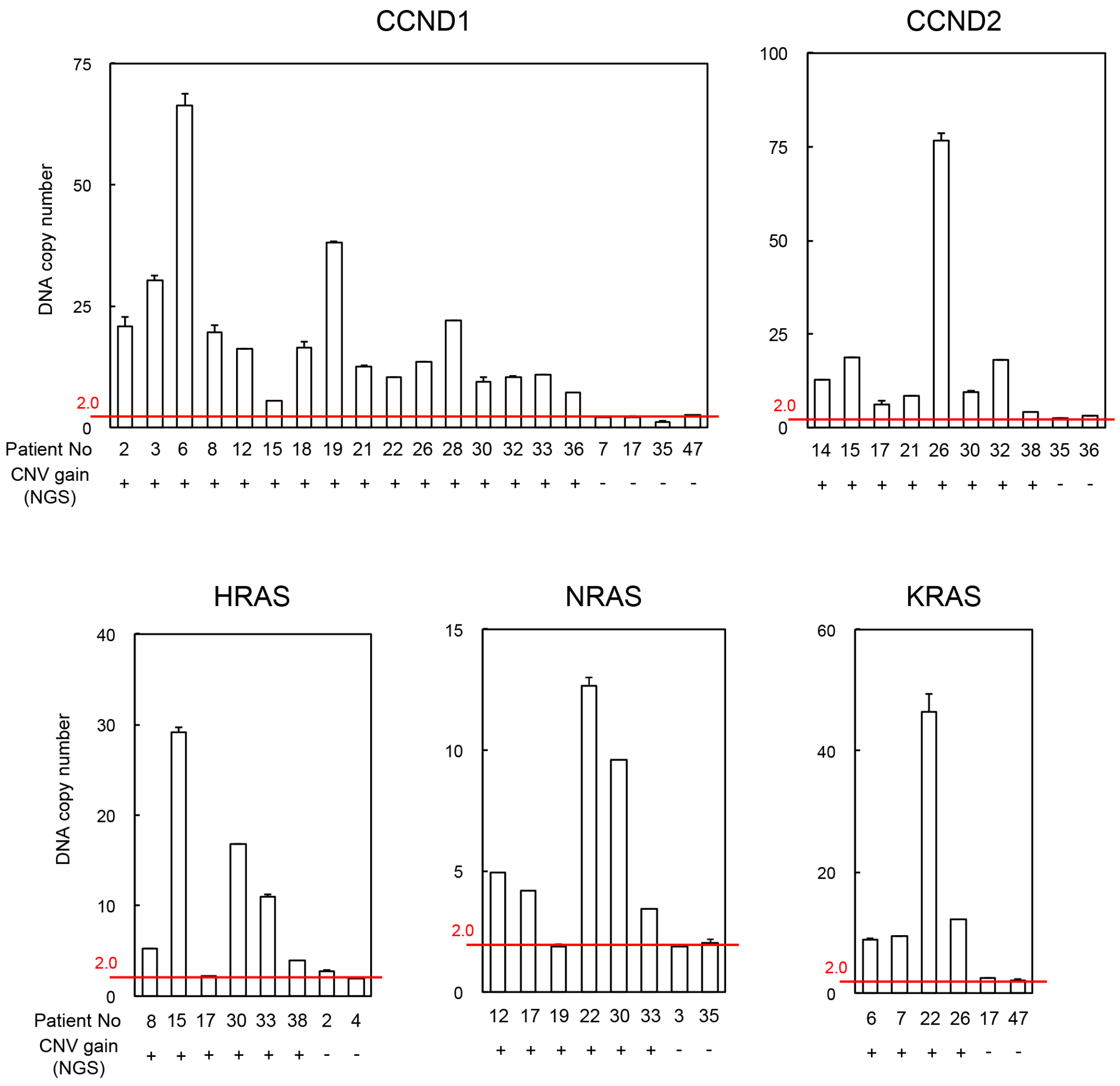

Supplementary Figure 10: qPCR copy number analysis. Relative DNA copy number was confirmed by quantitative real-time PCR using a KOD SYBR qPCR mix (Toyobo, Osaka, Japan) and the 7900HT real-time PCR system (Applied Biosystems). DNA content per haploid genome was normalized to that of a repetitive element, Line-1, and calculated by the comparative CT relative quantification method. DNA copy number relative to normal diploid leukocyte DNA was shown in Y axis. All assays were performed in triplicate. Copy number gains were considered when copy number was higher than 3.0 each gene. NGS results are presented at the bottom of the graph. The sequences of the primers used were as follows: *CCND1*, 5'- AATAAAAATTGCGGGTATTTTCTG-3' (sense) and 5'- TCCCACGAAACGCTACTTCT-3' (antisense); *CCND2*, 5'- CTGTTTCTGAGTCTCTGCAGTCT-3' (sense) and 5'- CCCACACTTCCAGTTGCGAT-3' (antisense); *HRAS* 5'- CCTCAGCCGAAAACCAAGATC-3' (sense) and 5'- CTTGAACATCCCAAATGCCAC-3' (antisense); *NRAS*, 5'- TTCTGGCATCAGTGAAACGC-3' (sense) and 5'- ATCTGTCCAAAGCAGAGGCAG-3' (antisense); *KRAS*, 5'- AGAGCTGGCACAGAGACCAAAC-3' (sense) and 5'- AGGCCTTTGGTATACGACCCA-3' (antisense); *Line-1* as internal control, 5'- AAAGCCGCTCAACTACATGG-3' (sense) and 5'- TGCTTTGAATGCGTCCCAGAG-3' (antisense).

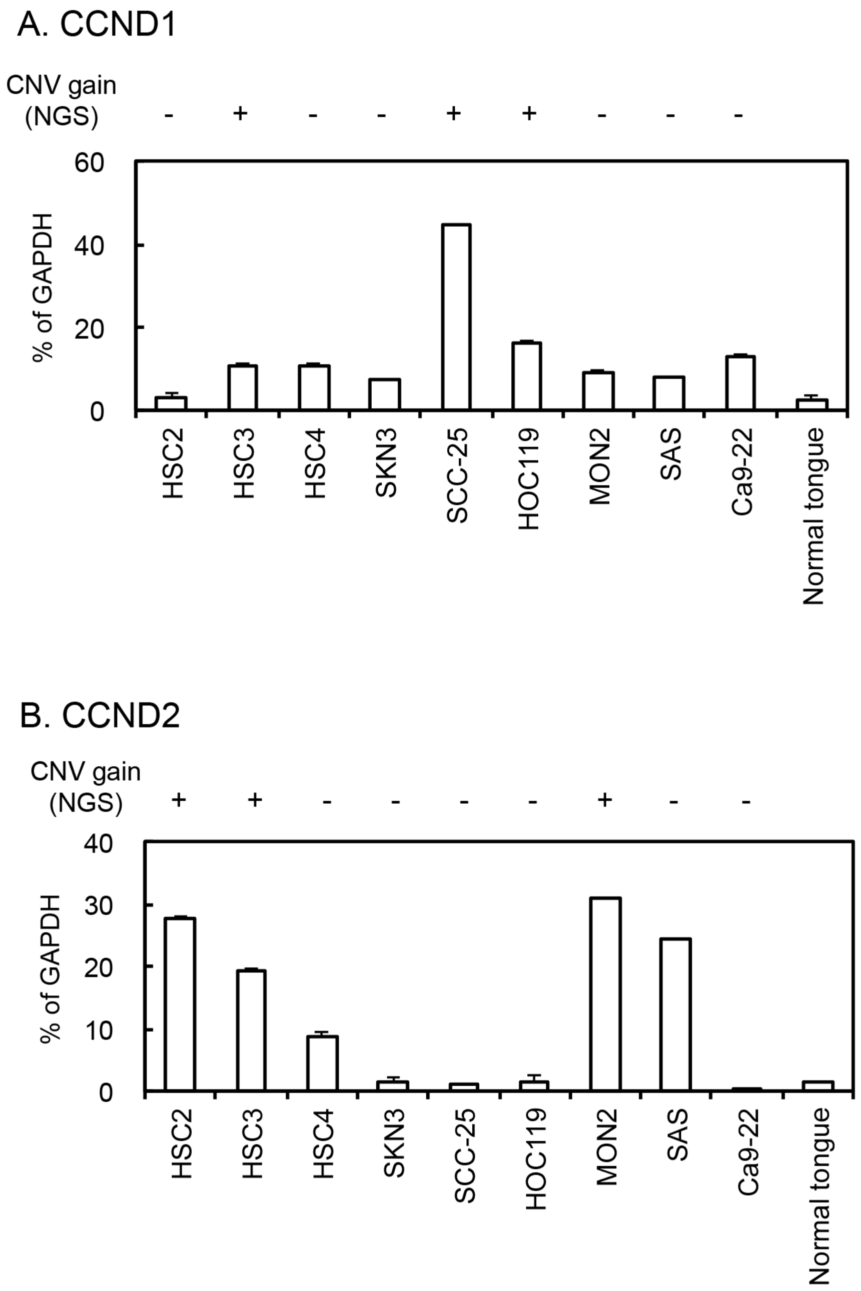

Supplementary Figure 11: Expression of endogenous *CCND1* and *CCND2* in a panel of cell lines. mRNA expression of *CCND1* **(A)** and *CCND2* **(B)** in OSCC cell lines was measured by real time RT-PCR using TaqMan Gene Expression assays and a 7900HT real-time PCR system (Applied Biosystems) according to the manufacturer's protocol. CNV results from NGS are presented at the top of the graph. The primer/probe sets used were as follows: *CCND1*, Hs00765553_m1; *CCND2*, Hs00153380_m1; and GAPDH, Hs99999905_m1. The data were normalized to the expression of the housekeeping gene *GAPDH* (% of GAPDH).

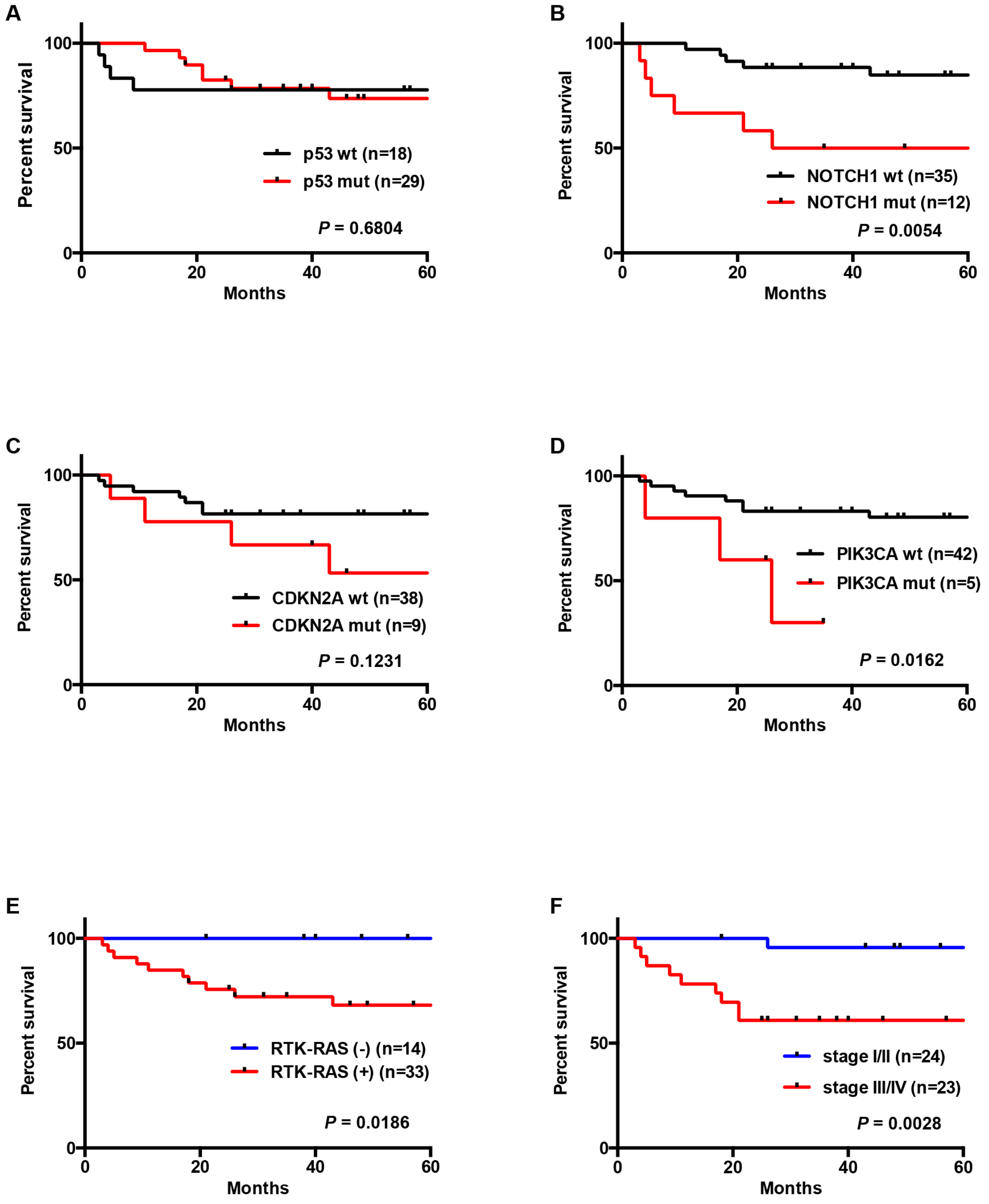

Supplementary Figure 12: Kaplan–Meier curves of overall OSCC patient survival. Kaplan–Meier estimates of overall survival among patients according to *TP53* mutation **(A)**, *NOTCH1* mutation **(B)**, *CDKN2A* mutation **(C)**, *PIK3CA* mutation **(D)**, RTK-RAS pathway alteration **(E)** and stage **(F)** (*P*, log-rank test). Analyses were carried out using Prism 6 (GraphPad Software, San Diego, CA, USA).

Supplementary Table 1: Summary of targeted amplicon sequencing data for OSCC

See Supplementary File 1

Supplementary Table 2: Full description of targeted amplicon sequencing data for OSCC tissues

See Supplementary File 2

Supplementary Table 3: Validated mutations by Sanger sequencing

See Supplementary File 3

Supplementary Table 4: Somatic nonsynonymous mutations found in OSCC cell lines

See Supplementary File 4

Supplementary Table 5: Somatic nonsynonymous mutations found in 47 OSCC

See Supplementary File 5

Supplementary Table 6: *NOTCH1* mutations detected in this study

See Supplementary File 6

Supplementary Table 7: List of somatic mutations in *CDKN2A and PIK3CA* genes

See Supplementary File 7
